# Thulium fiber laser: the new player for kidney stone treatment? A comparison with Holmium:YAG laser

**DOI:** 10.1007/s00345-019-02654-5

**Published:** 2019-02-06

**Authors:** Olivier Traxer, Etienne Xavier Keller

**Affiliations:** 1 Sorbonne Université, Service d’Urologie, Hôpital Tenon, Assistance-Publique Hôpitaux de Paris, 4 rue de la Chine, 75020 Paris, France; 2 Sorbonne Université, Groupe de Recherche Clinique sur la Lithiase Urinaire (GRC no 20), Hôpital Tenon, 75020 Paris, France; 3 Department of Urology, University Hospital Zurich, University of Zurich, Zurich, Switzerland

**Keywords:** Thulium fiber laser, Holmium:YAG laser, Lithotripsy, Urinary stone, Innovation

## Abstract

**Purpose:**

To compare the operating modes of the Holmium:YAG laser and Thulium fiber laser. Additionally, currently available literature on Thulium fiber laser lithotripsy is reviewed.

**Materials and methods:**

Medline, Scopus, Embase, and Web of Science databases were searched for articles relating to the operating modes of Holmium:YAG and Thulium fiber lasers, including systematic review of articles on Thulium fiber laser lithotripsy.

**Results:**

The laser beam emerging from the Holmium:YAG laser involves fundamental architectural design constraints compared to the Thulium fiber laser. These differences translate into multiple potential advantages in favor of the Thulium fiber laser: four-fold higher absorption coefficient in water, smaller operating laser fibers (50–150 µm core diameter), lower energy per pulse (as low as 0.025 J), and higher maximal pulse repetition rate (up to 2000 Hz). Multiple comparative in vitro studies suggest a 1.5–4 times faster stone ablation rate in favor of the Thulium fiber laser.

**Conclusions:**

The Thulium fiber laser overcomes the main limitations reported with the Holmium:YAG laser relating to lithotripsy, based on preliminary in vitro studies. This innovative laser technology seems particularly advantageous for ureteroscopy and may become an important milestone for kidney stone treatment.

**Electronic supplementary material:**

The online version of this article (10.1007/s00345-019-02654-5) contains supplementary material, which is available to authorized users.

## Introduction

The first use of Holmium:YAG laser in Urology was described more than two decades ago [1]. After having been evaluated as an innovative tool for tissue ablation with favorable hemostatic characteristics, the Holmium:YAG laser was eventually applied to urinary stones for lithotripsy [2]. Compared to other lithotripsy techniques, the Holmium:YAG laser presents several important advantages: (1) suitability for fragmentation of all known urinary stone types into small stone particles [3, 4]; (2) ability to operate with thin and flexible delivery fibers with limited energy losses and with core diameters as small as 200 µm [5, 6]; (3) favorable safety profile with minimal tissue penetration depth and low risk of undesirable tissue damage due to the relatively high absorption coefficient of the Holmium:YAG laser wavelength in water [7]; (4) versatility which allows a Holmium:YAG laser system to be used for soft tissue applications additionally to stones, which partially offsets the costs of high-power systems [8, 9].

Holmium:YAG laser has proved itself particularly beneficial for flexible ureteroscopy, where it has become the current gold standard for laser lithotripsy [6]. Laser generator parameters such as pulse energy and pulse frequency can be adapted by the operator [10, 11]. Urologists have shown a particular interest for low-pulse energy Holmium:YAG lithotripsy in recent years [12]. This setting seems to achieve particularly fine fragmentation of stones (“stone dust”) able to spontaneously evacuate, obviating the need for time-consuming retrieval of larger stone fragments [13–15]. To keep up with sufficient ablation rate, high-frequency Holmium:YAG generators have been developed for faster stone fragmentation with low-pulse energy settings [16]. Despite these innovations, the Holmium:YAG laser technology currently still faces limitations with regards to size of stones amenable to ureteroscopic laser lithotripsy [17–19].

Recently, another technology has been explored for next-generation laser lithotripsy: the Thulium fiber laser [20]. This promising technology offers several advantages over Holmium:YAG laser that may expand the boundaries of laser lithotripsy. The operating modes of both lasers are presented and compared in this article. Additionally, currently available literature on Thulium fiber laser is reviewed.

## Materials and methods

Literature on the operating modes of the Holmium:YAG and Thulium fiber lasers was reviewed. For systematic review of currently available evidence on Thulium fiber laser lithotripsy, a bibliographic search on Medline, Scopus, Embase, and Web of Science databases was conducted in October 2018. The search terms ‘Thulium fiber laser’ and ‘lithotripsy’ were used and the filters ‘English’ and ‘humans’ were applied. Only original articles were considered eligible. Supplementary Figure 1 shows a flow diagram summarizing the selection process. Owing to the heterogeneity of study outcomes, a narrative synthesis rather than a quantified meta-analysis of data was performed.

## Physical characteristics of Holmium and Thulium

### Holmium and Thulium ions

Holmium and Thulium are two distinct chemical elements with 67 and 69 protons in their nucleus, respectively, and have been classified as rare-earth elements in the periodic table. Holmium was first discovered by the Swiss chemists Marc Delafontaine and Jacques-Louis Soret in 1878 and was first named “Element X” [21, 22]. In 1879, the Swedish chemist Per Theodor Cleve observed a brown and a green substance while working on a sample of Erbium oxide. He named the brown substance Holmium (Holmia being the Latin name for Stockholm) and the green substance Thulium (after Thule, the place located furthest north in ancient Greek and Roman literature and cartography, thus referring to Scandinavia) [23]. Both Holmium and Thulium are predominantly found as trivalent ions in nature and in industrial applications such as lasers. Similar to other rare-earth ions, trivalent Holmium and Thulium ions have a unique set of emission wavelengths, particularly in the near-infrared range.

### Water absorption peak

**Fig. 1:**
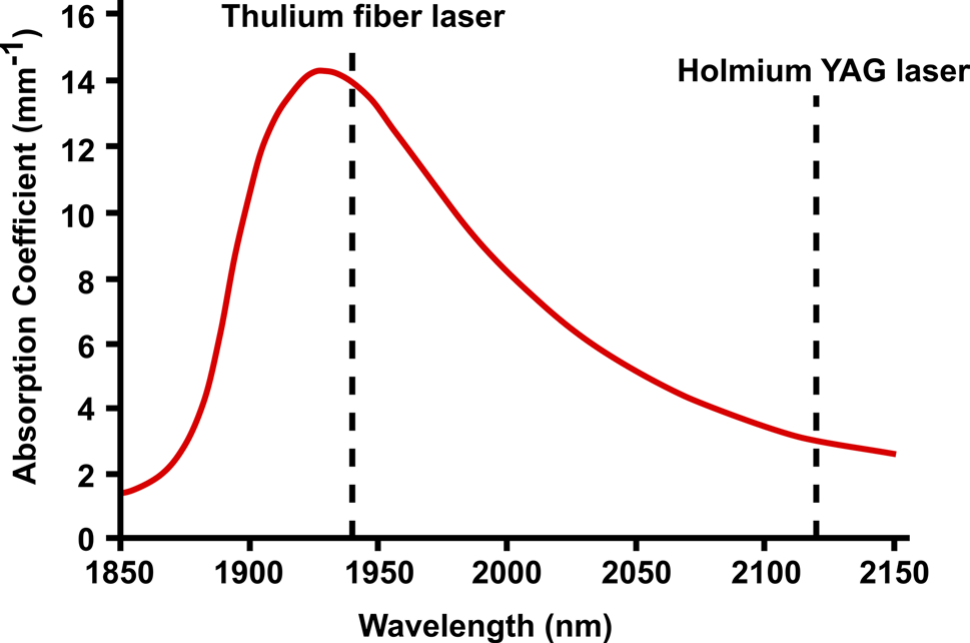
Absorption coefficient of liquid water at room temperature (22 °C) in the near-infrared range (red line). The Thulium fiber laser has been adapted to operate at 1940 nm, a wavelength close to a water absorption peak (approximatively 14 mm^−1^). Comparatively, the wavelength of the Holmium:YAG laser (2120 nm) has a much lower absorption coefficient in liquid water (approximatively 3 mm^−1^)

The near-infrared absorption peak of liquid water has been shown to be of particular relevance for laser–tissue interaction of Holmium- and Thulium-doped lasers (Fig. 1) [24]. The Holmium:YAG laser operates at 2120 nm and is highly absorbed in liquid water, leading to a rapid formation of a vapor bubble after emission in pulsed mode [25]. This interaction with water also adds to the safety profile of Holmium:YAG lasers, as the optical penetration depth is limited to 400 µm and coagulation of tissue beyond this distance only occurs in the high pulse energy range [7, 26]. Evidence of stone composition phase changes supports a photothermal interaction of Holmium:YAG laser with the stone matrix [4, 27, 28].

Multiple publications in the more general field of laser medicine also suggest other ablation mechanisms of hard tissue with predominant water absorption. Thermal expansion and vaporization of water are main mechanisms of hard tissue ablation for lasers with wavelengths close to infrared water absorption peaks such as 1940 and 2940 nm, where water is a primer laser chromophore [29–31] Although kidney stones are primarily of crystalline structure, these stones grow in a biological environment inside the body and have a complex microcrystalline composition, with intercrystalline spaces filled by water, often including a small but significant biological protein component in their structure as well [32]. Furthermore, multiple recent studies have reported on the porous structure of kidney stones, with intercrystalline spaces and pores observed at the small (nanometer) scale [33] up to the large scale (hundreds of micrometers) [34], sufficiently large for small water molecules to fill these intercrystalline spaces and pores. It is, therefore, also postulated that water present in intercrystalline spaces, pores, cracks, and fissures of human kidney stones undergo thermal expansion and vaporization during laser lithotripsy, thus contributing to the fragmentation of stones [35]. The thermal expansion coefficient of water is an order of magnitude higher than that for kidney stones with high pressure due to water vaporization contributing to this mechanism [36]. Recent scanning electron microscopy studies also show evidence of crack formation in kidney stones and partly unaltered crystalline composition of stone dust after laser lithotripsy, providing further evidence supporting this mechanism [4, 37].

For laser lithotripsy, the Thulium fiber laser has been optimized to emit at a wavelength of 1940 nm, thus closely matching the near-infrared absorption peak of liquid water at 22 °C (Fig. 1) [24]. Because the absorption coefficient of the Thulium fiber laser (approximately 14 mm^−1^) is more than four-fold higher than Holmium:YAG laser (approximately 3 mm^−1^), a lower threshold and higher ablation efficiency can be expected in favor of the Thulium fiber laser at equivalent pulse energies. A lower tissue and water penetration depth may potentially also add to the safety profile of the Thulium fiber laser.

Another advantage that is valid for both Holmium:YAG and Thulium fiber lasers is the possibility to transmit the laser beam through thin silica fibers. Silica fibers have favorable proprieties for their use in flexible ureteroscopy, allowing the transmission of the laser beam in fully deflected scopes [38].

## Characteristics of laser generators

### Holmium:YAG laser: an optical cavity with a solid-state crystal

**Fig. 2:**
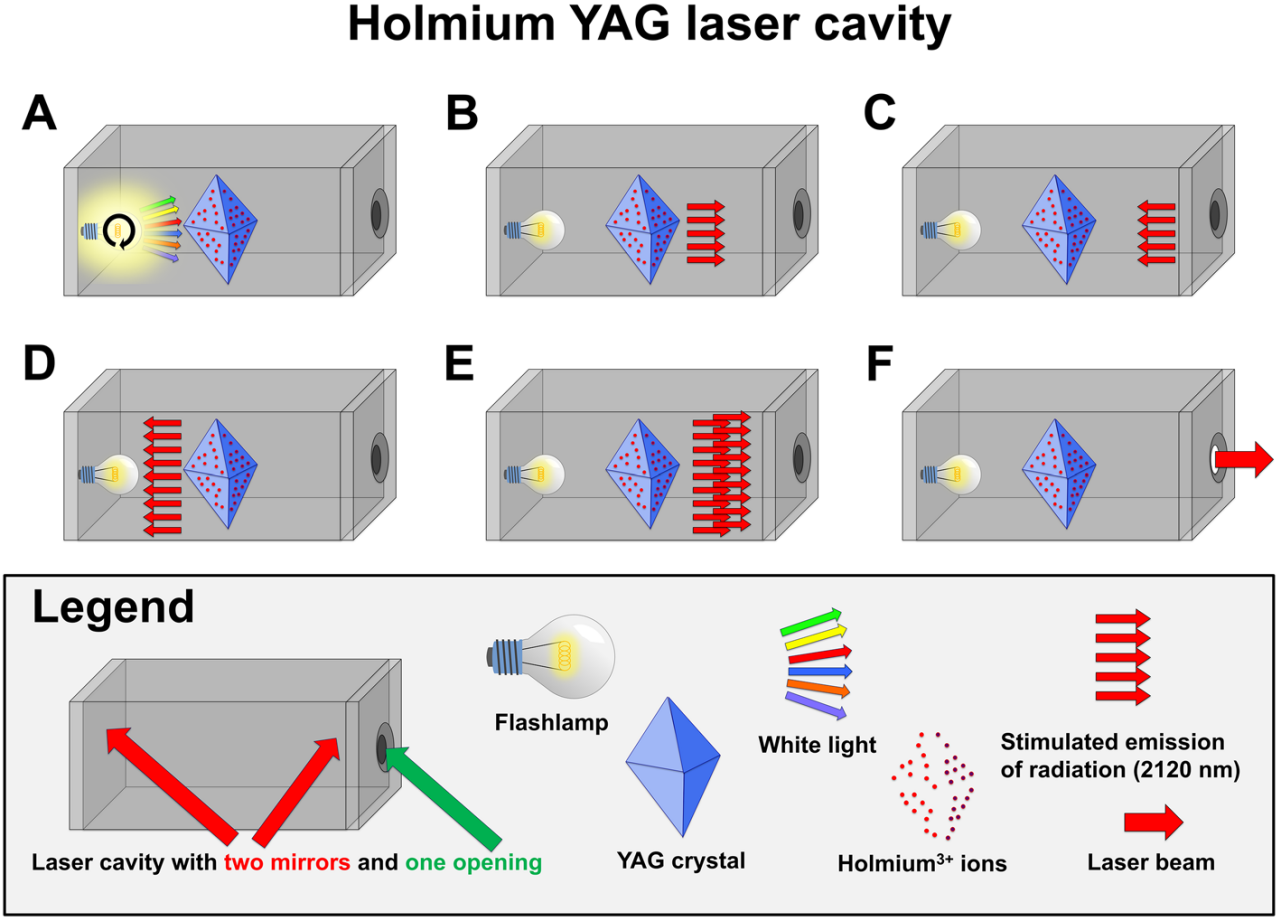
Schematic representation of the operating mode of a Holmium:YAG laser cavity. **a** Broad-spectrum white light is emitted from a flashlamp (typically Xenon or Krypton). **b** The white light interacts with the Holmium ions that are chemically bound to the YAG crystal and excites Holmium-electrons into higher-energy quantum states. **b** This interaction results in the emission of new photons with a characteristic wavelength of 2120 nm. Additional white light emitted from the flashlamp adds to Holmium ions excitation, a process referred to as “laser pumping”. **c** The radiation is reflected between the mirrors of the laser cavity. **d, e**: Because prior laser pumping excited numerous Holmium ions to higher-energy states, the reflected radiation will interact with the excited Holmium ions and stimulate emission of multiple additional photons at 2120 nm. This phenomenon is referred to as “light amplification by stimulated emission of radiation (LASER)”. **f** A transient opening of the cavity releases the radiation in the form of a pulsed laser beam

The Holmium:YAG laser beam originates from an optical cavity (Fig. 2). The central element of this cavity is a YAG crystal that has been chemically doped with Holmium ions. This architecture is referred to as a solid-state laser. For each laser pulse, the light emitted by a flashlamp (typically Xenon or Krypton) interacts with the Holmium ions and results in the emission of new photons with a characteristic wavelength of 2120 nm. These photons then travel freely within the optical cavity and are reflected by mirrors at each end of the cavity. Depending on the desired pulse energy, additional pump cycles can add to the energy of each single pulse. Finally, a small cavity opening allows the pulsed laser energy to exit the cavity when needed.

**Fig. 3:**
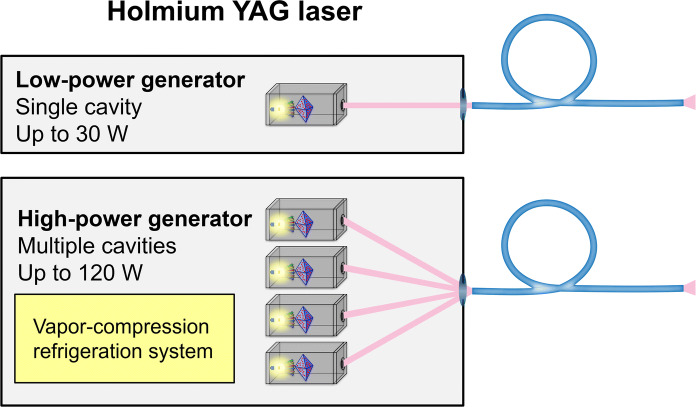
Schematic representation of Holmium:YAG laser generators. Low-power generators are made out of a single laser cavity (gray box) that emits its laser beam (pink) in line with the output connector and the proximal end of the laser fiber (blue). High-power generators incorporate multiple laser cavities (gray boxes) and require a complex alignment of laser beams (pink) to the output connector for safe transmission to the delivery fiber (blue). A vapor-compression refrigeration system (yellow box) is necessary for cooling of high-power Holmium:YAG generators

Most of the energy emitted by the flashlamp is wasted and causes the laser cavity to heat. This is caused by the fact that the flashlamp emits energy in a broad spectrum, whereas the Holmium:YAG system absorbs energy in a narrow spectral line, with overlap between the two not exceeding 7–8%. Therefore, Holmium:YAG laser generators require an adequate water-cooling system, contributing significantly to the large size of these generators. Of particular relevance, the maximal temperature range within the laser crystal sets a limit to the power and frequency at which a single Holmium:YAG cavity can operate (< 30 W, < 30 Hz). To palliate this limitation, Holmium:YAG generators with multiple cavities have been developed, allowing the advent of high-power (> 50 W) generators in recent years (Fig. 3).

Another limitation of the Holmium:YAG laser architecture is that the spatial beam profile of the output beam is multimodal, or non-uniform, with hotspots [39, 40]. This beam profile is more difficult to tightly focus down into a small spot, therefore typically limiting the use of the Holmium:YAG laser to optical fibers of 200 µm core diameter or larger [39].

Finally, the Holmium:YAG architecture is limited by its vulnerability to external shocks, which may result in a misalignment of the mirrors within the cavity and cause irreversible damage to the laser generator. Great care and attention are, therefore, required whenever manipulating or transporting a Holmium:YAG laser system.

### Thulium fiber laser: a chemically doped fiber

**Fig. 4:**
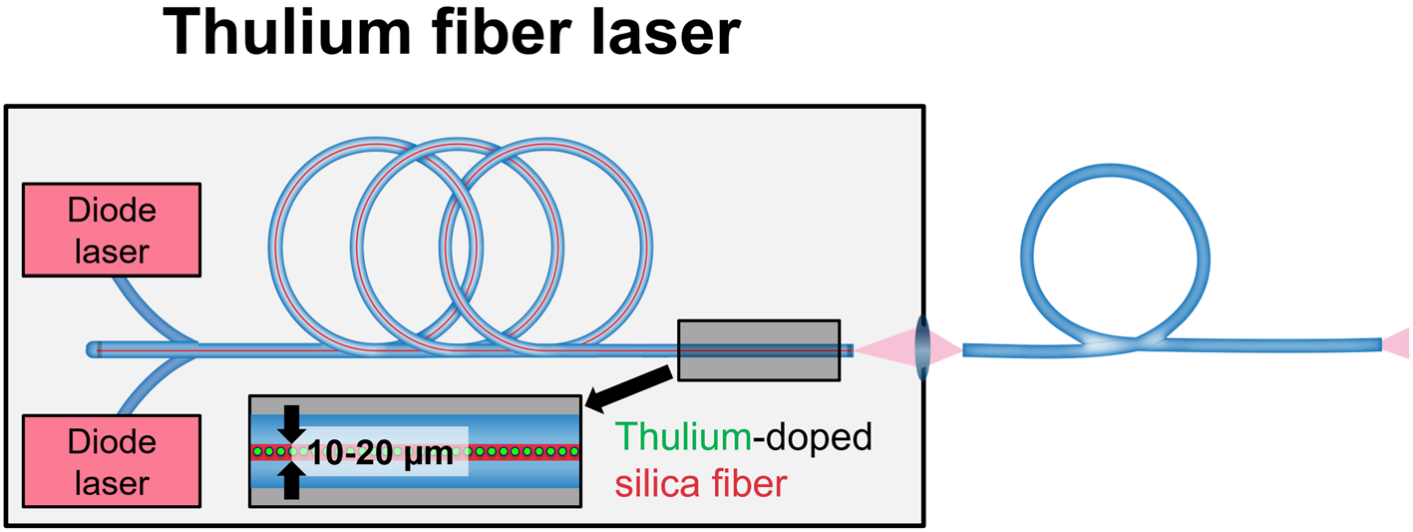
Schematic representation of a Thulium fiber laser. Laser pumping is achieved by electronically modulating diode lasers (pink boxes). A Thulium-doped, 10–20 µm core diameter, 10–30 m long silica fiber (red tube with green spots) is used as a gain medium for the generation of a laser beam. The uniform laser beam at the output connector allows for the use of laser fibers as small as 50 µm (blue)

As its name implies, the Thulium fiber laser consists of a very thin and long silica fiber (10–20 µm core diameter, 10–30 m long) which has been chemically doped with Thulium ions (Fig. 4). For laser pumping, multiple diode lasers are used to excite the Thulium ions. The emitted laser beam has a wavelength of 1940 nm and can operate either in a continuous mode or adopt a pulsed mode within a large range of various energy, frequency, and pulse shape settings (Table 1).

**Table 1:** Characteristics of two generators: Holmium:YAG laser and Thulium fiber laser

Parameter	Holmium:YAG laser (Lumenis Pulse 120H)	Thulium fiber laser (IPG Medical, Superpulse)
Wavelength	2120 nm	1940 nm
Pulse energy range	0.2–6.0 J	0.025–6.0 J
Pulse duration range	0.05–1 ms	0.05–12 ms
Pulse shape	Dictated by the pumping pulse	Electronically modulated
Maximum pulse frequency	120 Hz	2000 Hz
Maximum average power	120 W	60 W
Lowest proximal laser fiber core diameter	≥ 200 µm	≥ 150 µm
Cooling system	Low-power generators: self-contained water-cooling system with fan High-power generators: vapor-compression refrigeration system	Fan
Resistance to external shocks	Low	High

Efficiency of the fiber laser design is significantly higher than that of the flashlamp-pumped solid state Holmium:YAG laser, because the emission spectrum of the diode laser used for laser pumping precisely matches Thulium ions’ absorption line. Hence, the Thulium fiber laser requires less heat dissipation and can potentially operate at high-power ranges (> 50 W) and high-frequency ranges (up to 2000 Hz) with forced air (e.g., simple fan ventilation) inside the generator, compared to water-cooled Holmium:YAG lasers [41]. Also, the architecture of fiber lasers is insensitive to shock-related damages, unlike Holmium:YAG generators, because no mirror is involved in the fiber laser design.

The spatial beam profile of the laser beam emitted from a Thulium fiber laser, due to the small fiber core size in which the light originates, consists of only a few modes, and appears Gaussian in shape [40]. This more uniform spatial beam profile enables simpler focusing of the beam down to a very small spot for efficient coupling and transmission of high power through ultra-small fibers (e.g., 50–100 µm) [42].

Finally, it is important not to confuse the Thulium fiber laser with the Thulium:YAG laser. The former has a fiber laser construction and operates at 1940 nm, as opposed to the solid-state design of the Thulium:YAG laser (similar architecture to Holmium:YAG) which operates at 2010 nm. Therefore, any prior observations or clinical evaluations made with Thulium:YAG lasers cannot be directly applied to Thulium fiber lasers.

## Next-generation laser lithotripsy: what do we need?

From a historical point of view, it should be recalled that high-power, multiple-cavity Holmium:YAG laser generators have been primarily developed to meet the needs for ablative tissue applications such as Holmium enucleation of the prostate [8, 43]. It is only recently that the high-frequency range—and not the high-power range—of multiple-cavity Holmium:YAG generators has been proposed to offer advantages for laser lithotripsy. This is because stone dusting techniques for ureteroscopy—which require low-pulse energy and high frequency—have been gaining popularity in recent years [13–16, 44, 45]. Nevertheless, no study to date has been able to provide evidence for a substantial advantage of high-power Holmium generators over low-power generators for lithotripsy.

We herein present requirements that next-generation laser generators should meet to offer a real advantage for ureteroscopic laser lithotripsy.

### Smaller fibers

Prior studies on ureteroscopic Holmium laser lithotripsy have shown multiple advantages in favor of smaller laser fibers: better irrigation flow, better instrument deflection, and less stone retropulsion [46–49]. Another major potential advantage in favor of smaller fibers would be the possibility to reduce the working channel diameter of ureteroscopes, thus allowing for a major overall instrument miniaturization [50]. This would increase the space available between the ureteroscope and the ureter or access sheath, thus increasing irrigation outflow. The net result would be an overall increase of irrigation flow, higher irrigation turnover within renal cavities and most importantly better visibility.

One additional observation from an in vitro study on Holmium:YAG lithotripsy deserves particular attention: at equal laser settings, the smallest size of stone fragments was achieved by the smallest available fiber (272 µm core diameter) [49]. This observation was valid for both calcium oxalate monohydrate (COM) and uric acid (UA) stones and was found for all evaluated pulse energy levels (0.5, 1.0, and 1.5 J). An explanation may be that smaller fibers enable laser irradiation of a smaller area on stone surface, thus decreasing the probability for large fragments to detach from the initial stones.

Considering the above observation, a fiber size as small as possible would be desirable for laser lithotripsy. This is precisely a limitation of Holmium:YAG lasers; these generators can only safely accept fibers with a core diameter ≥ 200 µm. This is explained by the poorly focused multimode laser beam profile at the coupling interface between the laser generator and the proximal end of the delivery fiber, which increases the probability of generator and fiber damage by heat generation [39]. Comparatively, the Thulium fiber laser generates a much more uniform and focused laser beam, which can be transmitted to laser fibers with smaller core diameters (50–150) µm [40, 42]. Consequently, the Thulium fiber laser offers the potential for miniaturized next-generation ureteroscopy that may integrate remarkably thin fibers [51].

### Lower pulse energy

**Fig. 5:**
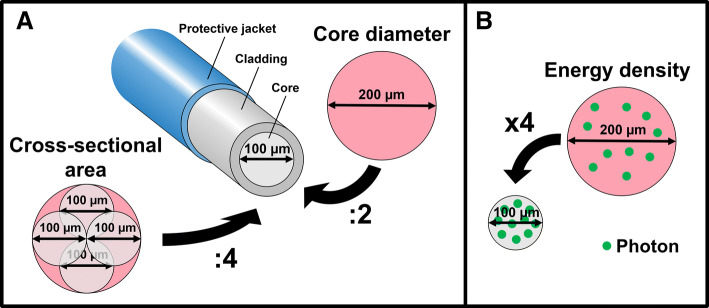
Relationship between fiber core diameter, cross-sectional area and energy density. **a** When the core diameter is divided by two, the cross-sectional area is divided by four. **b** When the core diameter is divided by two, the energy density is increased by four

A known limitation during the use of smaller fibers is the risk of fiber tip degradation at high pulse energy levels [52]. When the core diameter is divided by two, the energy density is increased by four (Fig. 5). Therefore, as a rule of thumb, pulse energy should be divided by four when the fiber core diameter is divided by two. Longer pulse duration may also add to the prevention of fiber tip degradation [53]. A third parameter that may arguably impact fiber tip degradation may be the temporal pulse profile (pulse shape in time), although this was not evaluated in any study yet.

In that respect, the Thulium fiber laser offers several potential advantages over Holmium:YAG laser. Notably, it can provide energy per pulse as low as 0.025 J, is capable of long-pulse duration (up to 12 ms) and emits a more uniformly shaped temporal beam profile (e.g., top-hat or flat-top) such that energy is more uniformly distributed across the duration of the pulse than the Holmium:YAG laser (Table 1) [54].

### Higher frequency

As detailed above, any decrease in laser fiber core diameter also requires a proportionate decrease in pulse energy. To keep up with stone ablation efficacy (amount of stone ablated over time), a compensatory increase in pulse repetition rate (frequency) is necessary.

Here again, the construct architecture of the Thulium fiber laser outperforms the Holmium:YAG laser, as pulse repetition rate can reach up to 2000 Hz, compared to the maximum of 80 Hz for current multiple-cavity Holmium:YAG laser generators (Table 1).

## Literature review

Table 2 summarizes findings of prior experimental studies comparing Holmium:YAG laser and Thulium fiber laser for lithotripsy. Multiple studies reported about a 1.5–4 times faster stone ablation rate in favor of the Thulium fiber laser, when lithotripsy was performed on COM or UA stones [55–57]. Of importance, limited rise of irrigation temperature up to 39 °C was found at high repetition rate (500 Hz) and low-pulse energy (0.035 J) in an in vitro ureter model [57]. As for coupling of the fiber to the laser generator, no damages to the proximal fiber end was found after Thulium fiber laser energy delivery (105 µm core diameter fibers), while all proximal fiber ends were damaged after Holmium lithotripsy (270 µm core diameter fibers) [58].

**Table 2:** Prior experimental studies comparing Holmium:YAG laser and Thulium fiber laser for lithotripsy

References	Year	Aim of the study	Study settings	Laser settings		Primary outcome	Secondary outcomes
				Holmium	Thulium		
Blackmon et al. [55]	2010	To compare lithotripsy efficiency between the Holmium:YAG laser and the Thulium fiber laser	100 µm core diameter fiber; lithotripsy on COM and UA stones	0.07 J 3 Hz 220 ms pulse duration	0.07 J 10 Hz 1000 µs pulse duration	5–10 times higher ablation efficiency in favor of the Thulium fiber laser	At 0.07 J, the Thulium fiber laser produces cleaner craters on stones at 1 ms compared to 20 ms pulse duration
Blackmon et al. [56]	2011	To compare ablation threshold and retropulsion between the Holmium:YAG laser and the Thulium fiber laser	200–270 µm core diameter; lithotripsy on COM, UA and PoP stones	0.03–0.55 J 10 Hz 30–500 µs pulse duration	0.005–0.035 J 10–400 Hz 500 µs pulse duration	4 times lower ablation threshold in favor of the Thulium fiber laser	Holmium: linear increase of stone retropulsion with pulse energy; Thulium: minimal retropulsion at 0.035 J and ≤ 100 Hz
Blackmon et al. [70]	2013	To compare the stone-suctioning effect of the Holmium:YAG laser with the Thulium fiber laser	272 µm core diameter fiber; lithotripsy on PoP stones	0.035–0.36 J 20 Hz 300 µs pulse duration	0.035 J 10–350 Hz 500 µs pulse duration	Stone-suctioning effect is possible	Better stone-suctioning in favor of the Thulium fiber laser
Hardy et al. [57]	2014	To compare lithotripsy efficiency and irrigation temperature between the Holmium:YAG laser and the Thulium fiber laser	Holmium: 272 µm core diameter fiber; Thulium: 100 µm core diameter fiber; lithotripsy on COM stones	0.6 J 6 Hz 350 µs pulse duration	0.035 J 150–500 Hz 500 µs pulse duration	1.5, 4.3, and 7.3 times faster lithotripsy in favor of the Thulium fiber laser at 150, 300 and 500 Hz	Mean peak irrigation temperature of 24 °C for Holmium:YAG lithotripsy and 33 °C, 33 °C and 39 °C for Thulium fiber laser lithotripsy at 150, 300 and 500 Hz
Wilson [58]	2016	To compare proximal fiber tip damage between the Holmium:YAG laser and the Thulium fiber laser	Holmium: 270 µm core diameter fiber after in vivo lithotripsy; Thulium: 105 µm core diameter fiber after firing in air	0.6 J 6 Hz 350 µs pulse duration	0.035 J 50–400 Hz 500 µs pulse duration	No damage after laser delivery with the Thulium fiber laser; micrometric damages on all fibers after Holmium lithotripsy	–

*COM* calcium oxalate monohydrate, *UA* uric acid, *PoP* plaster of Paris

Table 3 summarizes findings of more general prior experimental studies exploring operating characteristics of the Thulium fiber laser. In 2005, the first report on Thulium fiber laser lithotripsy adapted a continuous-wave generator to operate in a pulsed mode and demonstrated the feasibility of lithotripsy on COM and UA stones [59]. Thereafter, fibers with a core diameter as small as 50–150 µm were repeatedly reported to efficiently deliver Thulium fiber laser beam on urinary stones [40, 42, 54, 60–68]. Also, cumulative evidence from a series of studies on distal fiber tip design suggests the muzzle tip design for prevention of stone retropulsion during Thulium fiber laser delivery [60, 62, 63, 67, 68].

**Table 3:** Prior experimental studies exploring operating characteristics of the Thulium fiber laser

References	Year	Aim of the study	Study settings	Laser settings	Primary outcome	Secondary outcomes
Fried et al. [59]	2005	To explore the Thulium fiber laser in pulsed mode for lithotripsy	300 µm core diameter fiber	1.0 J × 10 Hz at 20,000 µs pulse duration	In pulsed mode, Thulium fiber laser is capable of lithotripsy of COM and UA stones	–
Scott et al. [40]	2009	To explore ≤ 200 µm core diameter fibers for Thulium fiber laser lithotripsy	Laser fibers with 100, 150, and 200 μm core diameters; lithotripsy on COM and UA stones	0.07 J × 10–30 Hz at 1000 µs pulse duration	No damage to 100, 150, and 200 μm fibers below 40, 60, and 80 W, respectively	Endoscope irrigation flow decreased by 26%, and 42% for 100 and 200 μm fibers, compared to empty working channel; much more uniform laser beam in favor of the Thulium fiber laser compared to Holmium:YAG laser
Blackmon et al. [60]	2010	To explore a new tapered distal laser fiber tip for Thulium fiber laser lithotripsy	150 µm core diameter fiber with a 300 µm distal diameter	0.07 J × 10 Hz at 1000 µs pulse duration	Lower fiber damage in favor of the tapered tip	No impact of the tapered tip design on stone ablation efficiency, scope deflection, and irrigation flow rates
Blackmon et al. [61]	2012	To explore electronic modulation for Thulium fiber laser lithotripsy	100 µm core diameter fiber; lithotripsy on COM and UA stones	0.035 J × micro-pulse mode or 0.035 J × standard pulse mode	2 times higher ablation efficiency in favor of the micro-pulse mode	Comparable fiber deterioration and stone retropulsion between micro-pulse and standard mode
Hutchens et al. [62]	2013	To explore a new detachable distal fiber tip for Thulium fiber laser lithotripsy	New construct of a detachable 300 µm core diameter distal fiber tip that can be attached to a conventional 150 µm core diameter fiber; lithotripsy on COM stones	0.03 J × 20 Hz at 500 µs pulse duration	The detachable distal tip is operable	Similar stone ablation rates compared to conventional tapered distal fiber tip
Hutchens et al. [63]	2013	To explore a new hollow steel at the distal fiber tip for Thulium fiber laser lithotripsy	150 µm core diameter fiber with a new construct of a 1-cm long steel tube that was glued to the distal tip; lithotripsy on COM stones	0.034 J × 150 Hz at 500 µs pulse duration	Significantly lower fiber deterioration in favor of the new hollow steel construct	Comparable stone ablation rates to conventional 150 µm core diameter fiber; increased stone retropulsion with the new hollow steel construct
Hardy et al. [64]	2014	To explore Thulium fiber laser lithotripsy at 500 Hz	100 µm core diameter fiber; lithotripsy on COM and UA stones; a nitinol basket is used	0.035 J × 500 Hz at 500 µs pulse duration	Lithotripsy at 500 Hz is feasible	–
Blackmon et al. [42]	2014	To explore a 50 µm core diameter fiber for Thulium fiber laser lithotripsy	50 µm core diameter fiber; lithotripsy on COM stones	0.035 J × 50 Hz at 500 µs pulse duration	Delivery of up to 15 W under extreme bending (5 mm radius)	Endoscope irrigation flow decreased by only 10% compared to empty working channel; up to 3 mm fiber deterioration at the distal tip after 2 min of lithotripsy
Wilson [65]	2015	To explore damages to a nitinol basket by the Thulium fiber laser	100 µm core diameter fiber; laser firing with varying working distance to nitinol wires	0.035 J × 50–500 Hz at 500 µs pulse duration	No nitinol damage at a distance ≥ 1 mm	–
Wilson [66]	2016	To explore a new 100 µm core diameter fiber with a distal ball-tip design for Thulium fiber laser lithotripsy	100 µm core diameter fiber with a 300 µm distal ball-tip design; lithotripsy on COM stones	0.035 J × 300 Hz at 500 µs pulse duration	Similar lithotripsy efficiency compared to conventional fibers	Rapid deterioration of the ball-tip design
Hardy et al. [54]	2016	To explore bubble formation at the distal fiber tip with the Thulium fiber laser	105 and 270 µm core diameter fibers; firing in saline	0.005–0.065 J × at 200–1000 µs pulse duration	Maximal bubble length of 1.2 and 1.1 mm for the 105 and 270 µm fibers, respectively	–
Hutchens et al. [67]	2017	To explore a new fiber muzzle brake at the distal fiber tip for Thulium fiber laser lithotripsy	100 µm core diameter fiber with a new muzzle brake tip construct; lithotripsy on COM stones	0.035 J × 300 Hz at 500 µs pulse duration	2-times lower stone retropulsion in favor of the new muzzle brake construct	No signs of distal tip fiber deterioration after lithotripsy with the new muzzle brake
Gonzales et al. [68]	2018	To characterize vapor bubble dynamics of five different distal fiber tip designs	100 and 170 µm bare fiber tip, 150–300 µm tapered fiber tip, 100 and 300 µm ball tip fiber tip, 100 and 340 µm hollow steel tip, as well as 100 and 560 µm muzzle brake tip design	0.0034 J at 500 µs pulse duration	Maximal bubble length and highest stone retropulsion with the hollow steel design	Minimal bubble length and lowest stone retropulsion with the muzzle brake design

*COM* calcium oxalate monohydrate, *UA* uric acid

An analysis of Thulium fiber laser bubble formation at the distal fiber tip revealed the formation of a bubble stream with multiple bubble expansions and collapses [54]. This phenomenon is reminiscent of the Moses effect, which has been first described in 1988 as a vapor channel resulting from water irradiation by laser and which leaves an open path with low absorption coefficient between the fiber tip and the stone surface [69]. Notably, a stone-suctioning effect of Thulium fiber laser has been demonstrated to be achievable under certain circumstances [70]. How this bubble stream may impact on lithotripsy remains to be detailed in future studies.

A limitation to this literature review is that all currently available evidence on Thulium fiber laser originates from in vitro studies performed in a single study center. Future studies on the clinical application of the Thulium fiber laser are needed.

## Conclusions

The innovative operating characteristics of the Thulium fiber laser suggest that this new technology has a significant potential for urinary stone treatment. Based on preliminary in vitro studies, the Thulium fiber laser surpasses Holmium:YAG laser in many aspects: (1) integration of smaller fibers with a core diameter as small as 50 µm; (2) pulse energy as low as 0.025 J; (3) super-high pulse repetition rate range up to 2000 Hz. These new standards may become particularly advantageous for ureteroscopy and open paths that were not been amenable to Holmium:YAG laser.

## Electronic supplementary material

Below is the link to the electronic supplementary material.

Supplementary material 1 (PNG 144 kb)
