# Supplementary figures and images for: Thulium fiber laser: the new player for kidney stone treatment? A comparison with Holmium:YAG laser
Source: World J Urol. 2019 Feb 6;38(8):1883–94. doi: 10.1007/s00345-019-02654-5 (PMC7363731; doi:10.1007/s00345-019-02654-5)

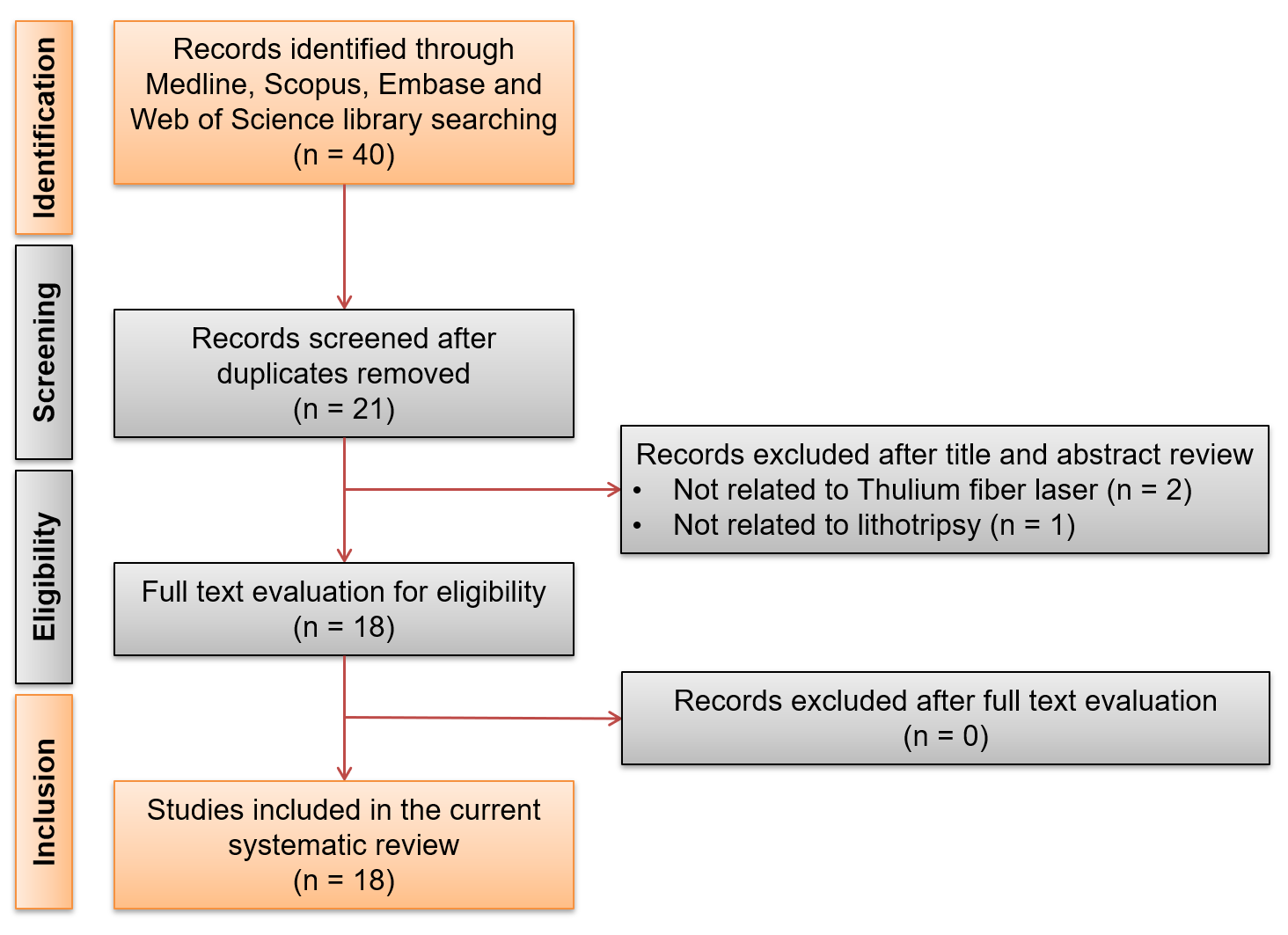

Supplement: Supplementary file 1 — Supplementary material 1 (PNG 144 kb) [file 345_2019_2654_MOESM1_ESM.png]
